# Associations of Dietary ω-3, ω-6 Fatty Acids Consumption with Sleep Disorders and Sleep Duration among Adults

**DOI:** 10.3390/nu13051475

**Published:** 2021-04-27

**Authors:** Jia Luo, Honghan Ge, Jing Sun, Kangyu Hao, Wenqin Yao, Dongfeng Zhang

**Affiliations:** Department of Epidemiology and Health Statistics, The School of Public Health of Qingdao University, No. 308 Ningxia Road, Qingdao 266071, China; luojia9605@163.com (J.L.); ghhlyy99@163.com (H.G.); sunjing1011@163.com (J.S.); haokangyu530@163.com (K.H.); yaowq18975098656@163.com (W.Y.)

**Keywords:** sleep disorders, sleep duration, dietary ω-3 fatty acids, dietary ω-6 fatty acids, dose–response relationship

## Abstract

The relationship between ω-3 and ω-6 fatty acids consumption and sleep disorders or duration are controversial. Therefore, we used the data of the National Health and Nutrition Examination Survey 2007–2016 in this cross-sectional study to explore their relationships. ω-3 and ω-6 fatty acids consumption was assessed using two 24 h dietary recall interviews. Sleep disorders and sleep duration were based on self-reported data. Logistic regression models and restricted cubic spline analyses were used. Compared with tertile one, the odds ratios (ORs) and 95% confidence intervals (CIs) of sleep disorders for the second tertile of ω-6 fatty acid intake and the highest tertile of ω-6:ω-3 ratio were 1.30 (1.04–1.62) and 1.36 (1.08–1.70), respectively. Inverse U-shaped and linear dose–response relationships were observed between dietary ω-6 fatty acid intake and ω-6:ω-3 ratio and sleep disorders, respectively. In addition, ω-3 fatty acid consumption was adversely related to sleep disorders in men and the OR (95% CI) was 0.68 (0.49–0.95). Compared with normal sleep duration, ω-3 fatty acid consumption was negatively related to very short, short, and long sleep duration risk. The relative risk ratios (RRRs) were 0.53 (0.35–0.81), 0.79 (0.67–0.93), and 0.81 (068–0.98), respectively. The RRR of very short sleep for ω-6 fatty acid consumption was 0.57 (0.45–0.73). Our study indicates that ω-6 fatty acid consumption and the ω-6:ω-3 ratio are positively associated with the risk of sleep disorders, while the negative association between ω-3 fatty acids and sleep disorders may exist only in men. Furthermore, ω-3 and ω-6 fatty acid consumption are negatively related to the risk of non-normal sleep duration.

## 1. Introduction

Sleep is necessary for health and plays a crucial part in physical and mental function, the immune system, and working or learning performance [1,2,3]. However, sleep disorders, which mainly include insomnia, parasomnias, hypersomnolence, circadian rhythm sleep-wake disorders, sleep-related movement and breathing disorders, and other sleep disorders [4], are common in adults with the prevalence ranges from 6.25% to 35.9% [5,6,7]. Specifically, sleep structure can differ depending on age and sex. The normal sleep process is affected by multiple factors with aging, manifesting as reduced sleep efficiency, increased sleep latency, and decreased deep slow-wave sleep [8]. These symptoms may also differ by sex [9,10]. Previous studies demonstrated that worse sleep condition (insufficient or excessive sleep duration as well as other sleep troubles) was related to adverse health outcomes, for example, metabolic disease, depression symptoms, cardiovascular disease, and cognitive function decline [11,12,13,14,15]. Therefore, it is urgent to explore the potentially modifiable risk factors of sleep disorders.

Epidemiologic studies have explored the relationship between sleep and dietary factors, such as vegetables and fruits [16], dairy [17], phytoestrogens [7], caffeine [18], and zinc [19]. As common and essential nutrients, polyunsaturated fatty acids (PUFAs) are mainly obtained from foods [20]. The dietary intake of PUFAs and their concentration in plasma or tissues is different by sex [21,22]. Previous studies found that ω-3 fatty acids might exert potential benefits on the cardiovascular system, mental health, and cognitive function [23]. Several studies have demonstrated that ω-3 fatty acids are essential for the functional maturation of the brain, and that ω-6 fatty acids are involved in inflammatory response regulation and the dynamics of sleep-inducing compounds (such as oleamide, lipid A, and prostaglandins), which might closely relate to sleep [24,25,26,27]. Although the parameters of sleep measurement were different, several studies indicated that a higher ω-3 fatty acids level was related to better sleep [28,29,30,31,32,33]. However, some studies did not find this positive association [34,35,36,37]. Few studies have investigated the association between ω-6 fatty acids and sleep, and the results were inconsistent. A recent cross-sectional study did not detect a significant association between erythrocyte membrane ω-6 fatty acids levels and sleep disorders in Chinese children and adolescents [32], whereas animal experiments have shown that prostaglandin D2 and E2 derived from arachidonic acid are very important sleep regulators [38,39]. In addition, the ratio of ω-6: ω-3 is commonly used to describe fatty acid composition. However, evidence is scarce on the association between the ratio of ω-6:ω-3 and sleep. After consideration of the inconsistent results and the unclear dose–response relationships, we conducted this present cross-sectional study using data from the National Health and Nutrition Examination Survey (NHANES) to explore the associations between dietary consumption of ω-6 and ω-3 fatty acids and sleep disorders and sleep duration.

## 2. Materials and Methods

### 2.1. Analytic Sample

We conducted this present cross-sectional study using the data from the National Health and Nutrition Examination Survey (NHANES), which is a continuous national survey conducted by the National Center for Health Statistics; data were released in 2 year cycles. More detailed information about NHANES has been described in previous studies [40,41].

The samples for sleep disorders (n = 18,310) and sleep duration (n = 21,153) were limited to participants from the NHANES 2007–2014 and 2007–2016, respectively, because the NHANES 2015–2016 did not include data about sleep disorders. We excluded those under 18 years old, with unreliable 24 h recall dietary data (dietary recalls status that did not meet the reliable and minimum standards), or missing sleep data. Then, we further excluded women who were lactating and pregnant and individuals with excessive energy intake (daily energy intake <500 kcal/d or >8000 kcal/d for men and <500 kcal/d or >5000 kcal/d for women). Participants using sedative-hypnotic drugs were also excluded (Figure S1).

### 2.2. Outcomes

We defined sleep disorders according to the self-reported doctor-diagnosis. Participants were asked the following question: “Have you ever been told by a doctor or other health professional that you have sleep disorders?” Participants who answered “Yes” were considered to have sleep disorders. Sleep duration was collected using the following question: “How much sleep do you usually get at night on weekdays or workdays?” and was further categorized as long (≥9 h), normal (7–<9 h), short (5–<7 h), and very short (<5 h) sleep duration [7,42].

### 2.3. Dietary ω-3 and ω-6 Fatty Acids Consumption

Dietary ω-3 and ω-6 fatty acids consumption was assessed by two 24 h dietary recall interviews. The detailed classification of ω-3 and ω-6 fatty acids is consistent with our previous work [41]. The daily consumption of ω-3 and ω-6 fatty acids was adjusted for energy intake [43].

### 2.4. Covariates

We included these variables as covariates to control the potential confounding effect: age, sex, annual household income, educational level, marital status, race/ethnicity, smoking status, caffeine intake, drinking status, hypertension, diabetes, depressive symptoms, body mass index, work-related physical activity, recreational physical activity, and sampling seasons. Detailed categories of covariates are presented in Table S1.

### 2.5. Statistical Analysis

Numbers (percentages) and medians (interquartile range) are used to describe qualitative and non-normal distributed data, respectively. Mann–Whitney U tests were adopted to compare the difference between participants with and without sleep disorders according to distribution characteristics. Moreover, to compare qualitative variables, chi-square tests are used. The energy-adjusted ω-3 and ω-6 fatty acids consumption (mg/kcal/d) and the ratio of ω-6:ω-3 were segmented into tertiles, with the lowest group (tertile 1) being the reference.

The associations of dietary ω-3 and ω-6 consumption and the ω-6:ω-3 ratio with sleep disorders were examined by binary logistic regression analyses. Only sex and age were adjusted in model 1. Model 2 further adjusted for annual household income, educational level, race/ethnicity, marital status, smoking status, drinking status, caffeine intake, hypertension, diabetes, depressive symptoms, body mass index, work-related physical activity, recreational physical activity, and sampling seasons. The dose–response relationship was evaluated by restricted cubic spline with three knots (the 10th, 50th, and 90th percentiles of dietary fatty acids consumption) in the multivariate-adjusted model 2. To assess the relationship between ω-3 and ω-6 fatty acids consumption and sleep duration, multinomial logistic regression models were performed, using normal sleep duration (7–<9 h) as the reference. Given differences in sleep conditions between sexes and age groups [44], we performed the stratified analysis by sex and age groups. To generate a nationally representative estimate, we weighted the analysis with NHANES weighting guidelines. In sensitivity analysis, we classified participants who took sedative-hypnotic medicines into the sleep disorders group to analyze the association between dietary PUFAs intake and sleep disorders, as they presented as the most likely group to have sleep disorders. Stata 15.0 (Stata Corporation, College Station, TX, USA) was utilized to perform all statistical analysis. A two-sided *p*-value less than 0.05 was recognized as statistically significant.

## 3. Results

Table 1 presents the characteristics of the study participants by sleep disorders. Of the 18,310 participants, women accounted for 50.95%, and the prevalence of sleep disorders was 8.36%. Compared with subjects without sleep disorders, participants with sleep disorders tended to be older, smokers, non-Hispanic white, more likely to have depressive symptoms, diabetic, and have lower household income, higher caffeine intake, and hypertension. Furthermore, participants with sleep disorders had a higher ω-6:ω-3 ratio and dietary ω-6 fatty acids consumption level.

Associations of dietary ω-3 and ω-6 fatty acids consumption and the ratio of ω-6:ω-3 with risk of sleep disorders are presented in Table 2. In the multivariate-adjusted model, compared with tertile one, the OR (95%CI) of sleep disorders for the second tertile consumption of ω-6 fatty acids was 1.30 (1.04–1.62). The second and third tertile ORs for ω-6:ω-3 ratios were 1.42 (1.13–1.78) and 1.36 (1.08–1.70), respectively. In men, the ORs of sleep disorders were 0.68 (0.49–0.95) and 1.82 (1.33–2.49) for the highest group of ω-3 fatty acids consumption and ω-6: ω-3 ratio, respectively. Additionally, the second tertile OR of dietary ω-6 fatty acids intake was 1.49 (1.10–2.02). However, no significant association was observed in women (Table S2). In the stratified analysis by age groups, for participants aged 18–44 years, intake of ω-3 fatty acids was inversely associated with sleep disorders (OR = 0.67, 95% CI: 0.46–0.97). For subjects aged 60 years or older, the OR of sleep disorders for the second tertile of dietary ω-6 fatty acids was 1.50 (1.00–2.24). The ratio of ω-6:ω-3 was also positively related to sleep disorders for participants aged less than 60 years. The corresponding ORs were 1.46 (1.06–2.02) and 1.70 (1.11–2.61) (Table S3). In the sensitivity analysis, the OR for sleep disorders in the second tertile dietary ω-6 fatty acids compared with the lowest tertile was 1.32 (1.07–1.63) in the fully adjusted model. The ORs for sleep disorders in the second and third tertiles of ω-6:ω-3 were 1.37 (1.14–1.66) and 1.35 (1.11–1.63), respectively. The sex-stratified analysis resulted in similar findings (Table S4). Sensitive analysis revealed that our results were stable.

**Figure 1:**
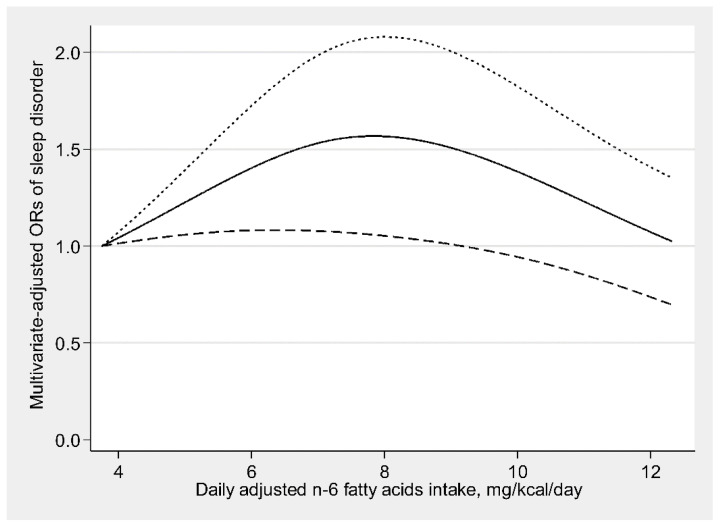
Restricted cubic spline model of the odds ratios (ORs) of sleep disorders with dietary ω-6 fatty acids consumption. The solid line and dashed lines represent the estimated ORs and the 95% confidence intervals.

**Figure 2:**
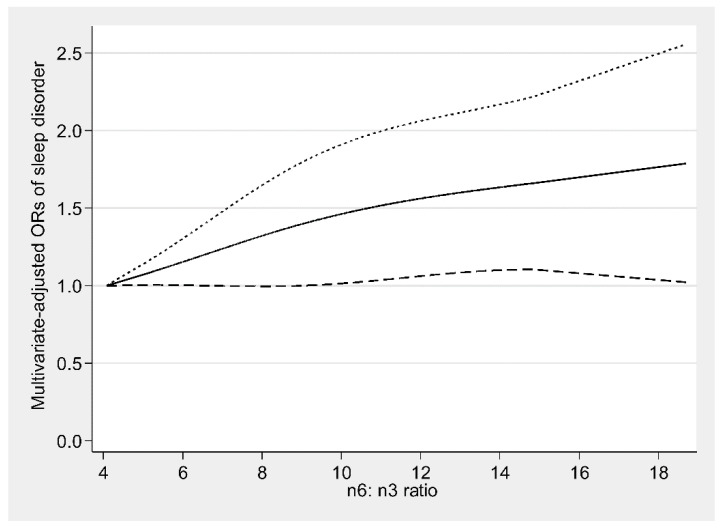
Restricted cubic spline model of the odds ratios (ORs) of sleep disorders with dietary ω-6:ω-3 ratio. The solid line and dashed lines represent the estimated ORs and the 95% confidence intervals, respectively.

**Figure 3:**
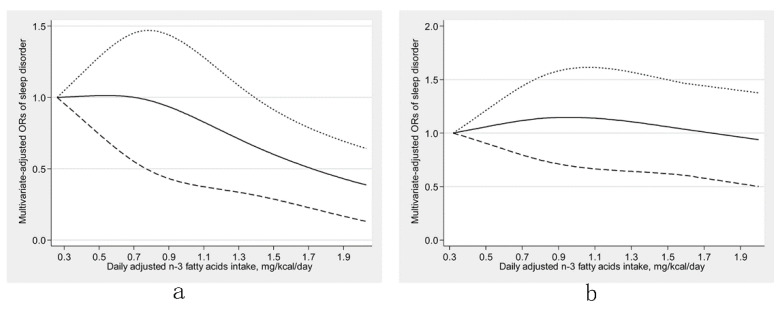
Restricted cubic spline models of the odds ratios (ORs) of sleep disorders with dietary ω-3 fatty acid consumption for men (**a**) and women (**b**). The solid line and dashed lines represent the estimated ORs and the 95% confidence intervals, respectively.

**Figure 4:**
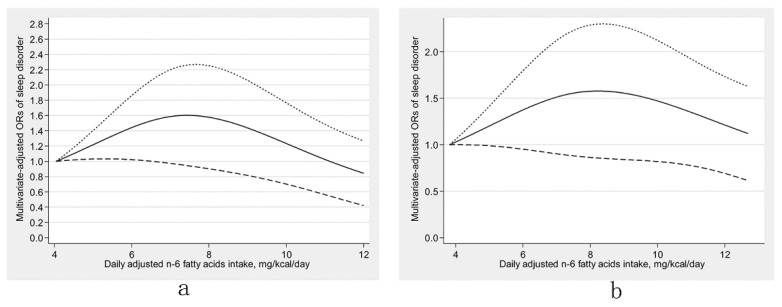
Restricted cubic spline models of the odds ratios (ORs) of sleep disorders with dietary ω-6 fatty acids consumption for men (**a**) and women (**b**). The solid line and dashed lines represent the estimated ORs and the 95% confidence intervals, respectively.

**Figure 5:**
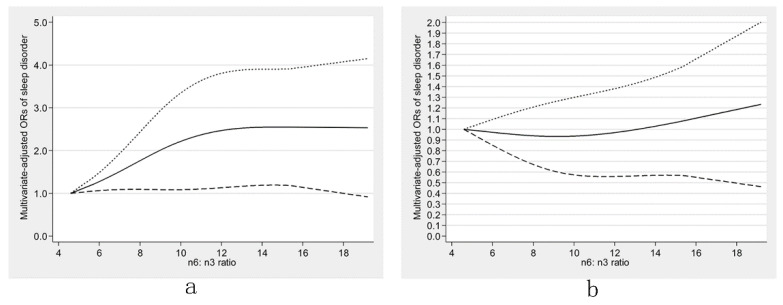
Restricted cubic spline models of the odds ratios (ORs) of sleep disorders with dietary ω-6:ω-3 ratio for men (**a**) and women (**b**). The solid line and dashed lines represent the estimated ORs and the 95% confidence intervals, respectively.

There was an inverse U-shaped relationship (P-nonlinearity = 0.002) between dietary ω-6 fatty acids consumption and sleep disorders (Figure 1). An approximately linear positive association (P-nonlinearity = 0.625) was found between ω-6:ω-3 and sleep disorders (Figure 2). We further explored the dose-response relationships in different sexes. In men, dietary ω-3 fatty acid consumption was inversely linearly related to sleep disorders (P-nonlinearity = 0.758) (Figure 3a). The dose-response relationships of both sexes are depicted in (Figure 3, Figure 4 and Figure 5).

Table 3 shows the association of dietary ω-3 and ω-6 fatty acids consumption as well as ω-6:ω-3 ratio with sleep duration. Compared with normal sleep duration, dietary ω-3 fatty acids consumption was negatively related to very short sleep, short sleep, and long sleep duration risk. The relative risk ratios (RRRs) with corresponding 95% CIs were 0.61 (0.46–0.80), 0.83 (0.73–0.95), and 0.81 (0.68–0.98) in model 2, respectively. Another negative correlation between dietary consumption of ω-6 fatty acids and very short sleep duration risk was observed (RRR = 0.57,95% CI: 0.45–0.73). No statistically significant association was observed between ω-6:ω-3 and sleep duration. Furthermore, dietary ω-3 fatty acids intake was adversely related to very short sleep (RRR = 0.53, 95% CI: 0.35–0.81) and short sleep duration (RRR = 0.79, 95% CI: 0.67–0.93) in men, but not in women. Dietary ω-6 fatty acid intake was also negatively related to very short sleep risk in both men (RRR = 0.53, 95% CI: 0.34–0.84) and women (RRR = 0.62, 95% CI: 0.45–0.85) (Table S5).

In the stratified analysis by age, dietary ω-3 fatty acids intake was inversely related to very short and short sleep for subjects aged 18 to 44 years. The corresponding RRRs with 95% CI were 0.59 (0.37–0.92) and 0.80 (0.67–0.96), respectively. Dietary intake of ω-3 fatty acids was also negatively associated with long sleep duration (RRR = 0.60, 95%CI: 0.42–0.87) for subjects aged 45 to 59 years. Dietary intake of ω-6 fatty acids was inversely correlated to the risk of very short sleep duration (RRR = 0.48, 95% CI: 0.34–0.69) in adults under 45 years (Table S6).

## 4. Discussion

According to our knowledge, this was the first study using a large and nationally representative sample to evaluate the relationships of dietary ω-3 and ω-6 fatty acids and the ratio of ω-6:ω-3 with sleep disorders as well as sleep duration in U.S. adults. Our research found that dietary ω-6 fatty acids consumption was related to sleep disorder risk in an inverse U-shaped manner and the ω-6:ω-3 ratio was positively linearly associated with the risk of sleep disorders. In men, similar results were observed and dietary ω-3 fatty acids intake was negatively linearly related to sleep disorders. Additionally, dietary ω-3 fatty acid consumption was negatively related to sleep disorders in middle-aged people, while the positive association between ω-6 fatty acid consumption and sleep disorders was found in older people. The ω-6:ω-3 was positively related to sleep disorders in people under 60 years old. In the stratified analysis by age or sex, negative associations between ω-3 and ω-6 fatty acid intake and non-normal sleep duration risk were observed.

Multiple epidemiological researchers have examined the relationship between ω-3 fatty acids or foods rich in ω-3 fatty acids (such as oily fish) and sleep. In observational studies, higher fish consumption was associated with better sleep quality [28,45,46]. Komada et al. [47] also found that the intake of fish and shellfish was correlated with ideal sleep duration in Japanese men. A previous study conducted in the U.K. indicated that high blood 22:6 ω-3 (DHA) concentration was related to better sleep and further DHA supplements significantly improved sleep duration and reduced awake episodes [29]. The conclusions of these previous studies are consistent with our findings. In contrast, results from an intervention study suggested that compared with the control group who ate meat, the experimental group who ate fish received no significant change in sleep onset latency, wake after sleep onset, and sleep duration [34]. Hansen et al. [37] also found no significant effects of fatty fish consumption on sleep quality.

The underlying mechanisms between ω-3 fatty acids and sleep are not fully established, and several potential explanations have been proposed. Firstly, ω-3 fatty acids are involved in maintaining nervous system function and intercellular signaling [48]. Dietary ω-3 fatty acids deficiency might influence the oscillatory activity of cortical neurons and the sleep-wake activity during sleep [24]. Second, previous animal research has found that a higher 22:6 ω-3 (DHA) intake was correlated to a higher 5-hydroxytryptamine (5-HT) concentration in the hippocampus [32,49]. 5-HT was considered as an essential substance in sleep preparation, triggering, and maintenance [50]. Third, poor sleep was associated with inflammation response [51]. The anti-inflammatory properties of ω-3 fatty acids might lower the risk of sleep disorders [25].

Direct evidence of the relevance between the dietary intake of ω-6 fatty acids and sleep is limited. This current study observed an inverse U-shaped dose-response relationship between ω-6 consumption and sleep disorder risk, which contradicts previous research by Tang et al. [32]. The mechanisms underlying the effects of dietary ω-6 fatty acids on sleep disorders remain unclear. One potential explanation is that ω-6 fatty acids serve as precursors of eicosanoids, a lipid mediator that shows a pro-inflammatory tendency [38,52], and inflammation response may increase the risk of sleep disorders [53].

The relationship between ω-6:ω-3 ratio and sleep has not been widely studied. Research conducted by Yehuda et al. [54] indicated that SR-3 (compounds comprising 1:4 ratios of ω-3:ω-6 fatty acids) supplements could improve the sleep quality of Alzheimer’s disease patients. The overall raising of the ω-6:ω-3 ratio resulted in decreased ω-3 fatty acid levels and increased arachidonic acid (AA) derived from ω-6 eicosanoids in tissues, including the brain [24,53], which may increase sleep disorder risk.

Relationships between dietary PUFA consumption and sleep duration were unclear. We provided several plausible explanations for this. Similar to how intermittent fasting improves cognitive function and insomnia symptoms, fatty acids can degrade to ketone bodies in the liver, and increased ketone bodies (e.g., β-hydroxybutyrate) in the brain might induce the transcription of brain-derived neurotrophic factors (BDNF), which might regulate sleep duration [55]. BDNF are also related to cognitive functioning [56] and further regulate sleep duration because short or long sleep duration is associated with poor cognitive functioning [57,58].

We observed substantial differences between sexes in the association between dietary fatty acid consumption and sleep disorder risk. One of the possible reasons for this is that several PUFAs in the body originate either from endogenous synthesis or from dietary sources. Estrogen might influence the enzyme involving the endogenous synthesis of PUFAs, leading to a higher level in women than men [21,59]. An increase in endogenous PUFA synthesis may reduce the effect of exogenous intake. Another reason may be the sex bias in the diagnosis of sleep disorders. In women, sleep disorders might be misdiagnosed and their prevalence is probably underestimated [60]. A potential explanation for observed age differences may be that sleep structure differs in different age groups [8]. The present study has several strengths. First, the use of a national survey with large sample size and rigorous quality control of NHANES ensured high generalizability. Second, we considered and controlled several potentially confounding factors. Third, the potential dose-response relationship was also researched. Finally, sex and age differences were also carefully assessed.

However, our results should be interpreted with caution and some limitations should be taken into account. First, our study was of a cross-sectional design; therefore, we could not make any causal inferences. Second, sleep disorders were based on self-reported doctor diagnoses rather than the international classification criteria for sleep disorders, which might lead to recall bias and reporting bias and may not be able to reflect objective sleep conditions. The sleep duration was also based on self-reported data rather than instrumental investigations. Third, limited data restrict the possibility to further research specific subtypes of sleep disorders. Fourth, we cannot rule out residual confounding, although many covariates were included in the multivariable model.

## 5. Conclusions

Our study suggested that dietary ω-6 fatty acids consumption and ω-6:ω-3 ratios were positively associated with the risk of sleep disorders. ω-3 fatty acids intake was negatively related to sleep disorders in men. Furthermore, dietary ω-3 fatty acids consumption was negatively related to very short, short, and long sleep duration. There was also an inverse correlation between dietary ω-6 fatty acids consumption and very short sleep duration.

## Figures and Tables

**Table 1:** Characteristics of participants by sleep disorders, NHANES 2007–2014 (n =18,310).

Characteristics	No Sleep Disorders	Sleep Disorders	*p* Value
Number of subjects (%) ^a^	16,779 (91.64)	1531 (8.36)	
Age (year) (%) ^a^			<0.001
18–44	7493 (44.61)	445 (29.07)	
45–59	3942 (23.47)	486 (31.74)	
≥60	5362 (31.92)	600 (39.19)	
Sex (%) ^a^			0.028
Male	8189 (48.81)	792 (51.73)	
Female	8590 (51.19)	739 (48.27)	
Race/ethnicity (%) ^a^			<0.001
Mexican American	2573 (15.33)	133 (8.69)	
Other Hispanic	1675 (9.98)	163 (10.65)	
Non-Hispanic white	7433 (44.30)	813 (53.10)	
Non-Hispanic black	3561 (21.22)	340 (22.21)	
Other races	1537 (9.16)	82 (5.36)	
Educational level (%) ^a^			0.041
<high school	4269 (25.47)	347 (22.66)	
High school	3914 (23.35)	359 (23.45)	
>high school	8579 (51.18)	825 (53.89)	
Annual household income (%) ^a^			<0.001
Below $20,000	3430 (21.31)	401 (27.06)	
$20,000 and over	12,667 (78.69)	1081 (72.94)	
Work physical activity (%) ^a^			0.303
Vigorous	3047 (18.16)	264 (17.25)	
Moderate	3629 (21.63)	314 (20.52)	
Other	10,099 (60.20)	952 (62.22)	
Recreational physical activity (%) ^a^			<0.001
Vigorous	3765 (22.44)	233 (15.22)	
Moderate	4527 (29.68)	371 (24.23)	
Other	8487 (50.58)	927 (60.55)	
Body mass index (%) ^a^			<0.001
<25 kg/m^2^	5244 (31.25)	225 (14.70)	
25 to <30 kg/m^2^	5526 (32.93)	362 (23.64)	
≥30 kg/m^2^	6009 (35.81)	944 (61.66)	
Marital status (%) ^a^			0.338
Married/Cohabitation	9439 (59.56)	879 (58.29)	
Windowed/Living alone	6410 (40.44)	629 (41.71)	
Depressive symptoms (%) ^a^	1532 (8.86)	361 (24.68)	<0.001
Diabetes (%) ^a^	2689 (16.03)	475 (31.03)	<0.001
Hypertension (%) ^a^	7933 (47.28)	977 (63.81)	<0.001
Smoke at least 100 cigarettes in life (%) ^a^	7017 (43.56)	665 (56.05)	<0.001
Had at least 12 alcohol drink a year (%) ^a^	11,150 (71.82)	1080 (73.67)	0.131
Caffeine intake (mg/d) ^b^	91 (167)	115 (195)	<0.001
Total energy(kcal/day) ^b^	1900 (1007)	1896 (941)	0.559
ω-3 fatty acid(mg/kcal/day) ^b^	0.77 (0.42)	0.77 (0.42)	0.836
ω-6 fatty acid(mg/kcal/day) ^b^	7.19 (3.32)	7.38 (3.41)	<0.001
ω-6: ω-3 ratio ^b^	9.08 (3.17)	9.23 (3.26)	0.001

Data are the number of subjects (percentage) or medians (interquartile ranges). ^a^ Chi-square test was used to compare the percentage between participants with and without sleep disorders. ^b^ Mann-Whitney U tests were used to compare the difference between participants with and without sleep disorders.

**Table 2:** Weighted odds ratios (95% confidence intervals) of sleep disorders across tertile of energy-adjusted dietary ω-3 and ω-6 fatty acid intake and ω-6:ω-3 ratio, NHANES 2007–2014.

	Cases/Participants	Crude	Model 1 ^a^	Model 2 ^b^
**Adjusted ω-3 (mg/kcal/d** **ay** **)**				
<0.65	514/6105	1.00 (ref)	1.00 (ref)	1.00 (ref)
0.65 to <0.91	501/6102	1.00 (0.84–1.19)	0.97 (0.81–1.16)	0.98 (0.80–1.21)
≥0.91	516/6103	0.91 (0.76–1.09)	0.84 (0.70–1.02)	0.85 (0.70–1.03)
**Adjusted ω-6 (mg/kcal/day)**				
<6.18	450/6104	1.00 (ref)	1.00 (ref)	1.00 (ref)
6.18 to <8.32	522/6103	1.19 (0.98–1.45)	1.18 (0.96–1.44)	1.30 (1.04–1.62) *
≥8.32	559/6103	1.15 (0.93–1.43)	1.09 (0.87–1.36)	1.07 (0.83–1.36)
**ω-6:ω-3 ratio**				
<8.19	451/6104	1.00 (ref)	1.00 (ref)	1.00 (ref)
8.18–10.15	535/6104	1.42 (1.17–1.73) **	1.48 (1.21–1.81) **	1.42 (1.13–1.78) **
≥10.15	545/6102	1.35 (1.12–1.64) **	1.42 (1.17–1.73) **	1.36 (1.08–1.70) **

Calculated using binary logistic regression models. ^a^ Model 1 adjusted for sex and age. ^b^ Model 2 adjusted for age, sex, race/ethnicity, educational level, annual household income, recreational physical activity, work physical activity, drinking status, smoking, hypertension, diabetes, depressive symptoms, body mass index, marital status, and sampling seasons. * *p* < 0.05; ** *p* < 0.01.

**Table 3:** Weighted relative risk ratios (95% CIs) for sleep duration (reference, 7–9 h/night) across tertiles of energy-adjusted dietary ω-3 and ω-6 fatty acid intake and ω-6:ω-3 ratio, NHANES 2007–2014.

	Model 2 ^a^		
	Very Short Sleep (<5 h/Night)	Short Sleep (5–<7 h/Night)	Long Sleep (≥9 h/Night)
**Adjusted ω-3 (mg/kcal/day)**			
<0.66	1.00 (ref)	1.00 (ref)	1.00 (ref)
0.66 to <0.93	0.79 (0.61–1.02)	0.98 (0.85–1.13)	0.81 (0.68–0.98) *
≥0.93	0.61 (0.46–0.80) **	0.83 (0.73–0.95) **	0.96 (0.78–1.18)
**Adjusted ω-6 (mg/kcal/day)**			
<6.27	1.00 (ref)	1.00 (ref)	1.00 (ref)
6.27 to <8.42	0.67 (0.52–0.87) **	0.92 (0.81–1.04)	0.88 (0.73–1.06)
≥8.42	0.57 (0.45–0.73) **	0.88 (0.77–1.01)	0.99 (0.82–1.21)
**ω-6:ω-3 ratio**			
<8.19	1.00 (ref)	1.00 (ref)	1.00 (ref)
8.18–10.15	0.99 (0.80–1.24)	1.06 (0.94–1.18)	0.80 (0.66–1.01)
≥10.15	1.08 (0.87–1.34)	1.07 (0.95–1.19)	0.90 (0.72–1.13)

Calculated using multinomial logistic regression models. ^a^ Model 2 adjusted for age, sex, race/ethnicity, educational level, annual household income, recreational physical activity, work physical activity, drinking, smoking, hypertension, diabetes, depressive symptoms, body mass index, marital status, and sampling seasons. * *p* < 0.05; ** *p* < 0.01.

## Data Availability

The datasets supporting the conclusions of this article are publicly available from the NHANES (https://www.cdc.gov/nchs/nhanes/index.htm).

## Supplementary Materials

The following are available online at https://www.mdpi.com/article/10.3390/nu13051475/s1, Figure S1: Flow chart of the screening process for the selection of eligible participants; Table S1: The classifications of categorical covariates; Table S2: Weighted odds ratios (95% confidence intervals) of sleep disorders across tertiles of energy-adjusted dietary ω-3, ω-6 fatty acid intake and ω-6:ω-3 ratios in fully adjusted model, stratified by sex, NHANES 2007–2014; Table S3: Weighted odds ratios (95% confidence intervals) of sleep disorders across tertiles of energy-adjusted dietary ω-3, ω-6 fatty acid intake and ω-6:ω-3 ratios in fully adjusted model, stratified by age, NHANES 2007–2014; Table S4: Weighted odds ratios (95% confidence intervals) of sleep disorders across tertiles of energy-adjusted dietary ω-3, ω-6 fatty acid intake and ω-6:ω-3 ratios in fully adjusted model, NHANES 2007–2014; Table S5: Weighted relative risk ratios (95% CIs) of sleep duration across tertiles of energy-adjusted dietary ω-3, ω-6 fatty acid intake and ω-6:ω-3 ratios in fully adjusted model, stratified by sex, NHANES 2007–2016; Table S6: Weighted relative risk ratios (95% CIs) of sleep duration across tertiles of energy-adjusted dietary ω-3, ω-6 fatty acid intake and ω-6:ω-3 ratios in fully adjusted model, stratified by age, NHANES 2007–2016.
