# Supplementary material for: Associations of Dietary ω-3, ω-6 Fatty Acids Consumption with Sleep Disorders and Sleep Duration among Adults
Source: Nutrients. 2021 Apr 27;13(5):1475. doi: 10.3390/nu13051475 (PMC8145923; doi:10.3390/nu13051475)
Supplement: Supplementary file 1 [file nutrients-13-01475-s001.zip › Supplementary File.pdf]

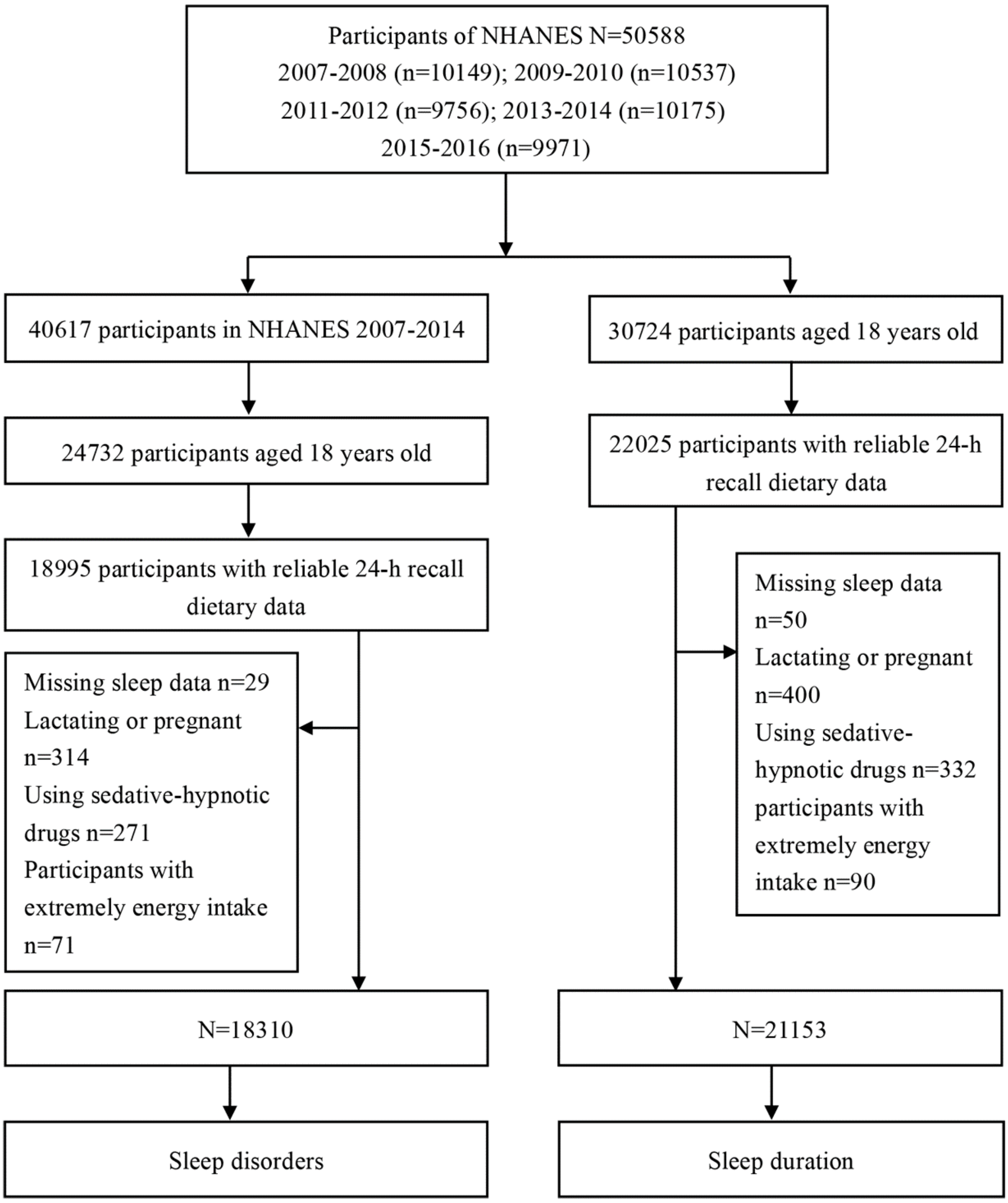

Figure S1. Flow chart of the screening process for the selection of eligible participants.

Table S1. The classifications of categorical covariates

Covariates	Classifications				
Age group	18-44 years,	44-59 years,	≥60years		
Gender	Males	Females			
Race/ethnicity	Mexican American,	Other Hispanic	Non-Hispanic White,	Non-Hispanic Black,	Other races
Marital status	Married/Living with partner,	Windowed/divorced/separated/Never married			
Educational level	Below high school	High school,	Above high school		
Annual household income	<$20000,	≥$20000,			
Body mass index	< 25 kg/m^2,	25 to <30 kg/m^2,	≥30 kg/m^2		
Work activity level	Vigorous activity,	Moderate activity,	Other		
Recreational activity level	Vigorous activity,	Moderate activity,	Other		
Smoking at least 100cigarettes in life	Yes,	No,			
Have at least 12 alcohol drinks/years	Yes,	No,			
Hypertension	Yes,	No,			
Diabetes	Yes,	No,			
Depressive symptoms	Yes,	No,			
Sampling seasons	November 1 through April 30	May 1 through October 31			
Caffeine intake	Continuous variable				
Energy intake	Continuous variable				

Table S2. Weighted odds ratios (95% confidence intervals) of sleep disorders across tertiles of energy-adjusted dietary ω-3, ω-6 fatty acid intake and ω-6: ω-3 ratios in fully adjusted model, stratified by gender, NHANES 2007-2014

	Male			Female		
	Crude	Model 1[a]	Model 2[b]	Crude	Model 1[a]	Model 2[b]
Adjusted ω-3 (mg/kcal/day)						
<0.65	1.00 (ref)	1.00 (ref)	1.00 (ref)	1.00 (ref)	1.00 (ref)	1.00 (ref)
0.65 to <0.91	1.01 (0.78-1.32)	0.97 (0.75-1.26)	0.98 (0.73-1.32)	1.00 (0.78-1.27)	0.96 (0.75-1.24)	1.00 (0.73-1.37)
⩾0.91	0.80 (0.61-1.07)	0.72 (0.54-0.97) *	0.68 (0.49-0.95) *	1.04 (0.82-1.35)	0.98 (0.75-1.27)	1.08 (0.81-1.44)
Adjusted ω-6 (mg/kcal/day)						
<6.18	1.00 (ref)	1.00 (ref)	1.00 (ref)	1.00 (ref)	1.00 (ref)	1.00 (ref)
6.18 to <8.32	1.44 (1.09-1.90) *	1.42 (1.07-1.88) *	1.49 (1.10-2.02) *	0.93 (0.69-1.24)	0.92 (0.68-1.24)	1.06 (0.77-1.46)
⩾8.32	1.16 (0.84-1.61)	1.08 (0.78-1.50)	1.01 (0.70-1.49)	1.10 (0.80-1.50)	1.04 (0.76-1.42)	1.10 (0.79-1.54)
ω-6: ω-3 ratio						
<8.19	1.00 (ref)	1.00 (ref)	1.00 (ref)	1.00 (ref)	1.00 (ref)	1.00 (ref)
8.18-10.15	1.53 (1.13-2.08) **	1.65 (1.20-2.27) **	1.72 (1.22-2.43) **	1.32 (1.00-1.74) *	1.37 (1.04-1.80) *	1.20 (0.89-1.63)
⩾10.15	1.66 (1.25-2.20) **	1.81 (1.36-2.40) **	1.82 (1.33-2.49) **	1.07 (0.81-1.42)	1.11 (0.84-1.47)	0.98 (0.70-1.37)

Calculated using binary logistic regression models. [a] Model 1 adjusted for age. [b] Model 2 adjusted for age, race/ethnicity, educational level, annual household income, recreational physical activity, work physical activity, drinking status, smoking, hypertension, diabetes, depressive symptoms, body mass index, marital status, sampling seasons. * p<0.05; ** p<0.01.

Table S3. Weighted odds ratios (95% confidence intervals) of sleep disorders across tertiles of energy-adjusted dietary ω-3, ω-6 fatty acid intake and ω-6: ω-3 ratios in fully adjusted model, stratified by age, NHANES 2007-2014

	18 ≤ Age < 45 Years			45 ≤ Age < 60 Years			Age ≥ 60 Years		
	Crude	Model 1[a]	Model 2[b]	Crude	Model 1[a]	Model 2[b]	Crude	Model 1[a]	Model 2[b]
**Adjusted ω-3 (mg/kcal/day)**									
<0.65	1.00 (ref)	1.00 (ref)	1.00 (ref)	1.00 (ref)	1.00 (ref)	1.00 (ref)	1.00 (ref)	1.00 (ref)	1.00 (ref)
0.65 to <0.91	1.09 (0.82-1.46)	1.10 (0.81-1.46)	1.34 (0.95-1.87)	0.96 (0.69-1.35)	0.96 (0.68-1.34)	0.89 (0.61-1.32)	0.82 (0.63-1.07)	0.83 (0.64-1.07)	0.81 (0.61-1.08)
⩾0.91	0.87 (0.60-1.25)	0.87 (0.61-1.26)	1.00 (0.68-1.48)	0.67 (0.46-0.98) *	0.67 (0.46-0.97) *	0.67 (0.46-0.97) *	0.97 (0.70-1.33)	1.00 (0.73-1.37)	0.95 (0.68-1.33)
**Adjusted ω-6 (mg/kcal/day)**									
<6.18	1.00 (ref)	1.00 (ref)	1.00 (ref)	1.00 (ref)	1.00 (ref)	1.00 (ref)	1.00 `(ref)	1.00 (ref)	1.00 (ref)
6.18 to <8.32	1.15 (0.81-1.65)	1.16 (0.81-1.66)	1.32 (0.90-1.94)	1.15 (0.84-1.60)	1.15 (0.84-1.57)	1.25 (0.86-1.81)	1.26 (0.92-1.71)	1.28 (0.94-1.74)	1.40 (0.99-1.97)
⩾8.32	1.04 (0.73-1.49)	1.05 (0.73-1.51)	1.06 (0.72-1.56)	0.83 (0.57-1.22)	0.83 (0.57-1.21)	0.85 (0.58-1.26)	1.51 (1.04-2.20) *	1.57 (1.09-2.26) **	1.50 (1.00-2.24) *
**ω-6: ω-3 ratio**									
<8.19	1.00 (ref)	1.00 (ref)	1.00 (ref)	1.00 (ref)	1.00 (ref)	1.00 (ref)	1.00 (ref)	1.00 (ref)	1.00 (ref)
8.18-10.15	1.54 (1.14-2.07) **	1.53 (1.14-2.06) **	1.46 (1.06-2.02) *	1.78 (1.23-2.58) **	1.79 (1.24-2.59) **	1.72 (1.17-2.54) **	1.15 (0.84-1.56)	1.16 (0.85-1.56)	1.09 (0.78-1.53)
⩾10.15	1.32 (0.98-1.79)	1.32 (0.97-1.78)	1.08 (0.75-1.54)	1.63 (1.09-2.43) *	1.63 (1.09-2.44) *	1.70 (1.11-2.61) *	1.42 (1.09-1.86) *	1.40 (1.08-1.85) *	1.30 (0.98-1.75)

Calculated using binary logistic regression. [a] Model 1 adjusted for gender. [b] Model 2 adjusted for gender, race/ethnicity, educational level, annual household income, recreational physical activity, work physical activity, drinking status, smoking, hypertension, diabetes, depressive symptoms, body mass index, marital status, sampling seasons. * $p<0.05$; ** $p<0.01$.

Table S4. Weighted odds ratios (95% confidence intervals) of sleep disorders across tertiles of energy-adjusted dietary ω-3, ω-6 fatty acid intake and ω-6: ω-3 ratios in fully adjusted model, NHANES 2007-2014

	Cases/Participants	Total participants[a]	Males[a]	Females[a]
**Adjusted ω-3 (mg/kcal/day)**				
<0.64	603/6193	1.00 (ref)	1.00 (ref)	1.00 (ref)
0.64 to <0.92	584/6189	0.92 (0.75-1.13)	0.75 (0.57-0.99) *	0.88 (0.64-1.22)
⩾0.92	606/6190	0.86 (0.72-1.03)	0.60 (0.42-0.86) **	0.96 (0.69-1.33)
**Adjusted ω-6 (mg/kcal/day)**				
<6.18	527/6191	1.00 (ref)	1.00 (ref)	1.00 (ref)
6.18 to <8.32	613/6191	1.32 (1.07-1.63) *	1.70 (1.25-2.31) **	1.23 (0.89-1.70)
⩾8.32	653/6190	1.06 (0.84-1.33)	1.48 (1.01-2.16) *	1.06 (0.69-1.61)
**ω-6: ω-3 ratio**				
<8.19	526/6191	1.00 (ref)	1.00 (ref)	1.00 (ref)
8.18-10.15	628/6191	1.37 (1.14-1.66) **	1.61 (1.18-2.19) **	1.22 (0.93-1.59)
⩾10.15	639/6191	1.35 (1.11-1.63) **	1.75 (1.29-2.36) **	1.03 (0.77-1.37)

Calculated using binary logistic regression models. [a]Models adjusted for age, gender, race/ethnicity, educational level, annual household income, recreational physical activity, work physical activity, drinking status, smoking, hypertension, diabetes, depressive symptoms,body mass index, marital status, sampling seasons. * $p<0.05$; ** $p<0.01$

Table S5. Weighted relative risk ratios (95% CIs) of sleep duration across tertiles of energy-adjusted dietary ω-3, ω-6 fatty acid intake and ω-6: ω-3 ratios in model 2, stratified by gender, NHANES 2007-2016

	Male			Female		
	Very Short Sleep (<5 h/Night)	Short Sleep (5- <7h/Night)	Long Sleep (≥9 h/Night)	Very Short Sleep (<5 h/Night)	Short Sleep (5- <7h/Night)	Long Sleep (≥9 h/Night)
Adjusted ω-3 (mg/kg/day)						
<0.66	1.00 (ref)	1.00 (ref)	1.00 (ref)	1.00 (ref)	1.00 (ref)	1.00 (ref)
0.66 to <0.93	0.67 (0.45-0.99) *	1.02 (0.85-1.22)	0.81 (0.62-1.07)	0.91 (0.65-1.29)	0.94 (0.77-1.15)	0.81 (0.62-1.06)
≥0.93	0.53 (0.35-0.81) **	0.79 (0.67-0.93) *	0.89 (0.64-1.23)	0.72 (0.52-1.01)	0.90 (0.75-1.07)	1.02 (0.80-1.31)
Adjusted ω-6 (mg/kg/day)						
<6.27	1.00 (ref)	1.00 (ref)	1.00 (ref)	1.00 (ref)	1.00 (ref)	1.00 (ref)
6.27 to <8.42	0.59 (0.39-0.88) **	1.03 (0.86-1.23)	0.92 (0.68-1.24)	0.77 (0.53-1.12)	0.81 (0.68-0.97) *	0.84 (0.66-1.08)
≥8.42	0.53 (0.34-0.84) **	0.90 (0.77-1.07)	0.89 (0.64-1.23)	0.62 (0.45-0.85) **	0.85 (0.70-1.03)	1.07 (0.82-1.39)
ω-6: ω-3 ratio						
<8.19	1.00 (ref)	1.00 (ref)	1.00 (ref)	1.00 (ref)	1.00 (ref)	1.00 (ref)
8.18-10.15	0.84 (0.61-1.17)	1.09 (0.93-1.27)	0.88 (0.66-1.18)	1.13 (0.77-1.66)	1.02 (0.86-1.22)	0.76 (0.59-1.01)
≥10.15	0.99 (0.71-1.39)	1.15 (0.97-1.36)	1.01 (0.73-1.39)	1.14 (0.82-1.58)	0.98 (0.85-1.13)	0.82 (0.63-1.07)

Calculated using multinomial logistic regression models. Model 2 adjusted for age, race/ethnicity, educational level, annual household income, recreational physical activity, work physical activity, drinking status, smoking, hypertension, diabetes, depressive symptoms, body mass index, marital status, sampling seasons. * p<0.05; ** p<0.01

Table S6. Weighted relative risk ratios (95% CIs) of sleep duration across tertiles of energy-adjusted dietary ω-3, ω-6 fatty acid intake and ω-6: ω-3 ratios in model 2, stratified by age, NHANES 2007-2016

	Model 2[a]		
	Very Short Sleep (<5 h/Night)	Short Sleep (5- <7h /Night)	Long Sleep (≥9 h/Night)
18 ≤ Age < 45 Years			
Adjusted ω-3 (mg/kcal/day)			
<0.66	1.00 (ref)	1.00 (ref)	1.00 (ref)
0.66 to <0.93	0.59 (0.41-0.83) **	0.99 (0.80-1.22)	0.94 (0.73-1.22)
≥0.93	0.59 (0.37-0.92) *	0.80 (0.67-0.96) *	1.21 (0.91-1.61)
Adjusted ω-6 (mg/kcal/day)			
<6.27	1.00 (ref)	1.00 (ref)	1.00 (ref)
6.27 to <8.42	0.47 (0.31-0.71) **	0.99 (0.83-1.18)	0.92 (0.71-1.20)
≥8.42	0.48 (0.34-0.69) **	0.89 (0.74-1.06)	1.13 (0.84-1.52)
ω-6: ω-3 ratio			
<8.19	1.00 (ref)	1.00 (ref)	1.00 (ref)
8.18-10.15	1.00 (0.61-1.63)	1.08 (0.90-1.30)	0.80 (0.55-1.15)
≥10.15	0.98 (0.68-1.41)	1.08 (0.90-1.28)	0.88 (0.60-1.30)
45 ≤ Age < 60 Years			
Adjusted ω-3 (mg/kcal/day)			
<0.66	1.00 (ref)	1.00 (ref)	1.00 (ref)
0.66 to <0.93	1.00 (0.64-1.55)	1.01 (0.78-1.31)	0.67 (0.46-0.96) *
≥0.93	0.72 (0.45-1.15)	0.91 (0.73-1.14)	0.60 (0.42-0.87) **
Adjusted ω-6 (mg/kcal/day)			
<6.27	1.00 (ref)	1.00 (ref)	1.00 (ref)
6.27 to <8.42	0.98 (0.68-1.55)	0.92 (0.72-1.17)	0.71 (0.46-1.09)
≥8.42	0.70 (0.45-1.08)	0.90 (0.69-1.18)	0.69 (0.43-1.13)
ω-6: ω-3 ratio			
<8.19	1.00 (ref)	1.00 (ref)	1.00 (ref)
8.18-10.15	1.23 (0.80-1.88)	1.08 (0.86-1.36)	0.88 (0.61-1.28)
≥10.15	1.09 (0.74-1.62)	1.00 (0.82-1.22)	0.96 (0.67-1.37)
Age ≥ 60 Years			
Adjusted ω-3 (mg/kcal/day)			
<0.66	1.00 (ref)	1.00 (ref)	1.00 (ref)
0.66 to <0.93	0.93 (0.60-1.43)	0.94 (0.73-1.21)	0.82 (0.58-1.15)
≥0.93	0.55 (0.36-0.84) **	0.83 (0.66-1.05)	1.00 (0.72-1.38)
Adjusted ω-6 (mg/kcal/day)			
<6.27	1.00 (ref)	1.00 (ref)	1.00 (ref)
6.27 to <8.42	0.81 (0.52-1.24)	0.80 (0.64-1.01)	0.97 (0.71-1.32)
≥8.42	0.64 (0.40-1.02)	0.84 (0.67-1.04)	1.07 (0.80-1.42)
ω-6: ω-3 ratio			
<8.19	1.00 (ref)	1.00 (ref)	1.00 (ref)
8.18-10.15	0.77 (0.49-1.20)	1.03 (0.85-1.24)	0.77 (0.59-1.01)
≥10.15	1.40 (0.81-2.40)	1.15 (0.92-1.43)	0.86 (0.66-1.12)

Calculated using multinomial logistic regression models. [a] Model 2 adjusted for gender, race/ethnicity, educational level, annual household income, recreational physical activity, work physical activity, drinking status, smoking, hypertension, diabetes, depressive symptoms, body mass index, marital status, sampling seasons. * $p<0.05$; ** $p<0.01$.
